# Variability of Practice, Information Processing, and Decision Making—How Much Do We Know?

**DOI:** 10.3389/fpsyg.2021.639131

**Published:** 2021-02-19

**Authors:** Stanisław H. Czyż

**Affiliations:** ^1^Faculty of Physical Education and Sport, University School of Physical Education in Wrocław, Wrocław, Poland; ^2^Faculty of Sport Studies, Masaryk University, Brno, Czechia; ^3^Physical Activity, Sport and Recreation Research Focus Area, North-West University, Potchefstroom, South Africa

**Keywords:** variability of practice, specificity of practice, information processing, motor learning, decision making, practice conditions

## Abstract

Decision-making is a complex action requiring efficient information processing. Specifically, in movement in which performance efficiency depends on reaction time, e.g., open-loop controlled movements, these processes may play a crucial role. Information processing includes three distinct stages, stimulus identification, response selection, and response programming. Mainly, response selection may play a substantial contribution to the reaction time and appropriate decision making. The duration of this stage depends on the number of possible choices an individual has to “screen” to make a proper decision. Given that reaction time is crucial in many sports, the possibilities of reducing it through practice are very tempting. The information processing and its relationship to the manner an individual is practicing are discussed. Especially the variability of practice issues will be explored. In variable practice conditions, an individual has to react to one or more stimuli and has to produce one of the many variations of the same movement or different movements they learned. One has to identify a stimulus appropriately and has to select a response optimally, i.e., choosing from as few choices as possible to reduce the reaction time. On the other hand, in constant practice conditions, an individual can be exposed to one or many stimuli. Still, there is only one variation of the movement that can be executed in the presence of a learned stimulus. Based on the information processing theory and the results of the research focusing on variability of practice, I discuss how the practice conditions may affect reaction time and, as a result, the decision-making process. I conceptually frame the possible implications of practice conditions on decision making related to information processing. In this review, a possible mechanism and relationship between practice conditions and decision-making are presented. Future research directions are presented.

## Introduction

When we practice a motor skill, we usually do it in the practice conditions that will prepare us for the future situations optimally. The optimum in this case means that we want to be prepared either for an unexpected non-trained situation or we would like to perform the acquired skill without any changes in its execution. In the former example, we aim at transfer of the acquired skill into a new situation. In the latter example, we want to stabilize the movement and perform it without any changes in its kinetic and spatio-temporal structure.

We may organize our practice manipulating many learning conditions: schedule (randomization), amount and distribution of practice, introducing part vs. entirety learning, or reducing the amount of feedback, etc. We may even choose between different learning approaches, e.g., observational, discovery or self-regulated learning, to prepare us better for future situations. There are already a number of studies focusing on the mechanisms and benefits and disadvantages of each approach and condition manipulation. Researchers have also identified how condition manipulation utilizes cognitive resources. Scheduling practice or randomization is one of the examples of such interest (e.g., Lee et al., 1994; Lee and Simon, 2004; Lee, 2012; Wright and Kim, 2020). Similarly, many studies were conducted on feedback and its facilitating role in motor learning (Anderson et al., 2020).

However, we know very little about what are the mechanisms responsible for the different benefits and disadvantages of variability in practice. We do not know much either about information processing and decision-making involved in variable and constant condition practice. Therefore, it is crucial to synthesize already existing knowledge and frame it for future research directions. A critical question is whether practice in variable conditions affects information processing and decision-making process differently than in constant conditions. I attempt to answer this question reviewing already existing literature.

At first, the term “variability of practice” is defined. The lack of common terminology, interchangeable use of the term in different contexts and meanings, and differing views is the reason it has to be defined at first. Secondly, we need to theoretically frame the phenomenon of “variability.” Two perspectives, i.e., schema theory and dynamic systems theory, will serve as a conceptual frame for further consideration. The information processing will be used as a base for further analysis. However, I will not focus on decision making processes related to the anticipation problem. These issues were quite comprehensively and exhaustively discussed in recent publications (Abernethy et al., 2012; Williams and Jackson, 2019).

## Variability of Practice and Variability in Performance

The term variability is used in two contexts. The first one relates to the variability in movement performance. It is also called trial-to-trial variability. When used in such context, the term variability means that movements belonging to the same class of action are never performed identically. There are differences between trials due to inherent motor noise, i.e., trial-to-trial variability in the execution of the movement (Dhawale et al., 2017). This issue is specifically exploited by neuroscientists as the trial-to-trial variability is considered an undesirable consequence of a noise in the motor system. This noise is related to the amount of force produced, i.e., the noise increases as the amount of force increases (Schmidt et al., 1979). The relationship between noise in the motor system and amount of force produced is linear (Schmidt et al., 1978). Trial-to-trial variability or variability in motor performance decreases while practice progresses (Deutsch and Newell, 2004), but it is never eliminated (Cohen and Sternad, 2009). The term variability used in such context is not related to the practice condition itself. The variability in performance is usually tested in very stable, predictable, and in as similar as possible (constant) trial-to-trial conditions (Cohen and Sternad, 2009). The theories explaining variability in performance are usually dynamical system theories, e.g., Bernstein's (1967, 1982), ecological (Gibson and Gibson, 1955; Gibson and Pick, 2000; Gibson, 2014), or Newell's dynamical systems theories (Newell, 1985, 1991).

**Figure 1:**
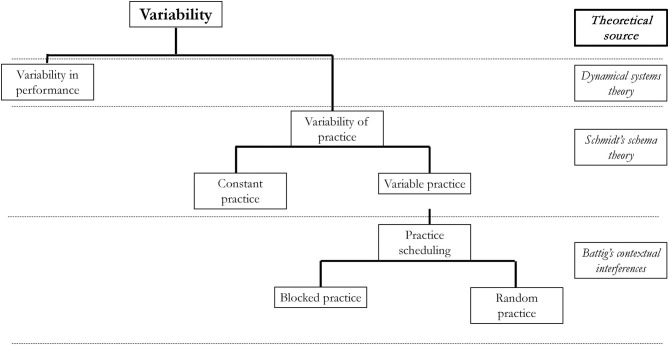
The context the “*variability of performance*” or “*variability of practice*” terms can be used.

The second context term “variability” is used in in motor learning is variability of practice or variable practice conditions. Again, the meaning of this term may vary depending on the context. Originally, it was one of the consequences or ideas drawn from the Schmidt' schema theory (Schmidt, 1975, 2003; Van Rossum, 1990; Sherwood and Lee, 2003). Variability of practice was used to explain the motor learning process. Motor learning was a function of variability in practice (Boyce et al., 2006). The term variability is also used when discussing practice scheduling or order of practice. The term variability in such context means random practice and it relates to the contextual interference phenomenon (Battig, 1966, 1972; Shea and Morgan, 1979). Although it makes sense to talk about variability of practice in random conditions (in high contextual interference), it may confuse people whether high contextual interference is the same as variable practice. Moreover, some authors use them interchangeably. The possible demarcations between these terms are shown in Figure 1.

## Theoretical Framework

The variability of practice was one of the key elements of Schmidt's schema theory. Dynamic theories were able to explain variability of practice benefits and disadvantages too, however, they were much better in explaining variability in performance as a consequence of the three learning stages recognized in Bernstein's theory, i.e., freezing, freeing, and exploiting (Huys et al., 2004).

### Schema Theory

Richard A. Schmidt developed his theory around two major assumptions (Schmidt, 1975, 1985, 2003). Firstly, he assumed that there is a motor program stored in memory. It is called Generalized Motor Program (GMP) and it is responsible for producing movements which belong to the same class (or category) of movements (Schmidt et al., 2018). GMP is described by the amount of force and spatial and temporal patterns of movement. It is specified by invariant features, i.e., patterns that are completely fixed from movement to movement, namely, relative force, relative timing, and order of sequences (Schmidt, 1985; Schmidt et al., 2018). There are also patterns of action that can be easily changed to control the force, sequencing and timing of movement. These are called parameters. By manipulating the parameters, an individual can adjust movement to a given situation. The relationship between parameters and outcomes is called recall schema (Sherwood and Lee, 2003; Boyce et al., 2006). Schmidt's theory assumed that individuals acquire motor skills by developing schemata. As the learning progresses, one knows more relationships between parameter assignment and an outcome, and, as a result, the schema is better established. And *vice versa*, the better is the schema developed, the more efficiently an individual can behave in a new situation. In other words, variable practice, i.e., learning relationships between parameters and outcomes, should enhance learning (Boyce et al., 2006). Moreover, individuals who practice more relationships should be better prepared for a novel situation. The simplest way of making individuals learn more relationships between parameters and outcomes is to make them assign different parameters to achieve the same or different goal. This type of practice is called “variable practice” as opposed to “constant practice,” i.e., practicing only one relationship between a set of parameters and one outcome. Sue Moxley termed this implication of the schema theory “the variability of practice hypothesis” (Moxley, 1979).

The variability of practice hypothesis was tested in many researches and many contexts, albeit not conclusively supporting the theory implication that performance on a novel task will be better following variable practice as opposed to the constant (Van Rossum, 1987, 1990). The biggest inconsistency between research results and theory implications was about the age of the learners (Shapiro and Schmidt, 1982; Van Rossum, 1987, 1990). Studies in children supported the variability of practice hypothesis more often, whereas research in adults were at least equivocal, and in some cases contradictory (Shapiro and Schmidt, 1982; Van Rossum, 1987). It was argued that the variability of practice hypothesis refers to the schema formation (development) rather than schema attainment, i.e., parameters assignment to the already existing motor program (Kerr, 1982; Van Rossum, 1987). Therefore, research should focus on a novel task and naïve participants to exclusively deal with schema development. The assumption behind this recommendation (Schmidt, 1975) was based on the conjecture that children have fewer schemata than adults, and this is why research on children more often supports the variability of practice hypothesis.

However, the review of studies utilizing children yielded equivocal results too, a few studies supported, and a few were contradictory to the variability of practice hypothesis (Van Rossum, 1990). Another explanation for the equivocality of the findings was provided by Lee et al. (1985). They suggested that it may be due to the scheduling of learned and tested tasks. Consequently, a distinction between random and blocked practice should be made. It directly refers to the contextual interference effect.

### Contextual Interference

Contextual interference (CI) effect has been originally described by Battig (1966), although the first study in motor learning was conducted by Shea and Morgan (1979). Battig reported that the way the practice is scheduled affects differently immediate performance and retention and transfer. When the practice was organized in so-called “random order,” i.e., it consisted of the performance of multiple motor skills in a random and rapidly changing order, it was defined as high CI. When the practice was organized (scheduled) in “blocked order,” it also consisted of the performance of multiple motor skills, however, each skill was repeatedly practiced until the number or time/number of trials allocated to that skill was used and it was practiced prior to the introduction of the next skill. The blocked order was defined as a low CI. Battig (1966) noted that high CI hinders performance during acquisition time, i.e., performance during practice is worse compared to the low CI practice. However, the high CI practice facilitates retention. Participants learning in high CI conditions had better retention results compared to the low CI practice groups.

**Figure 2:**
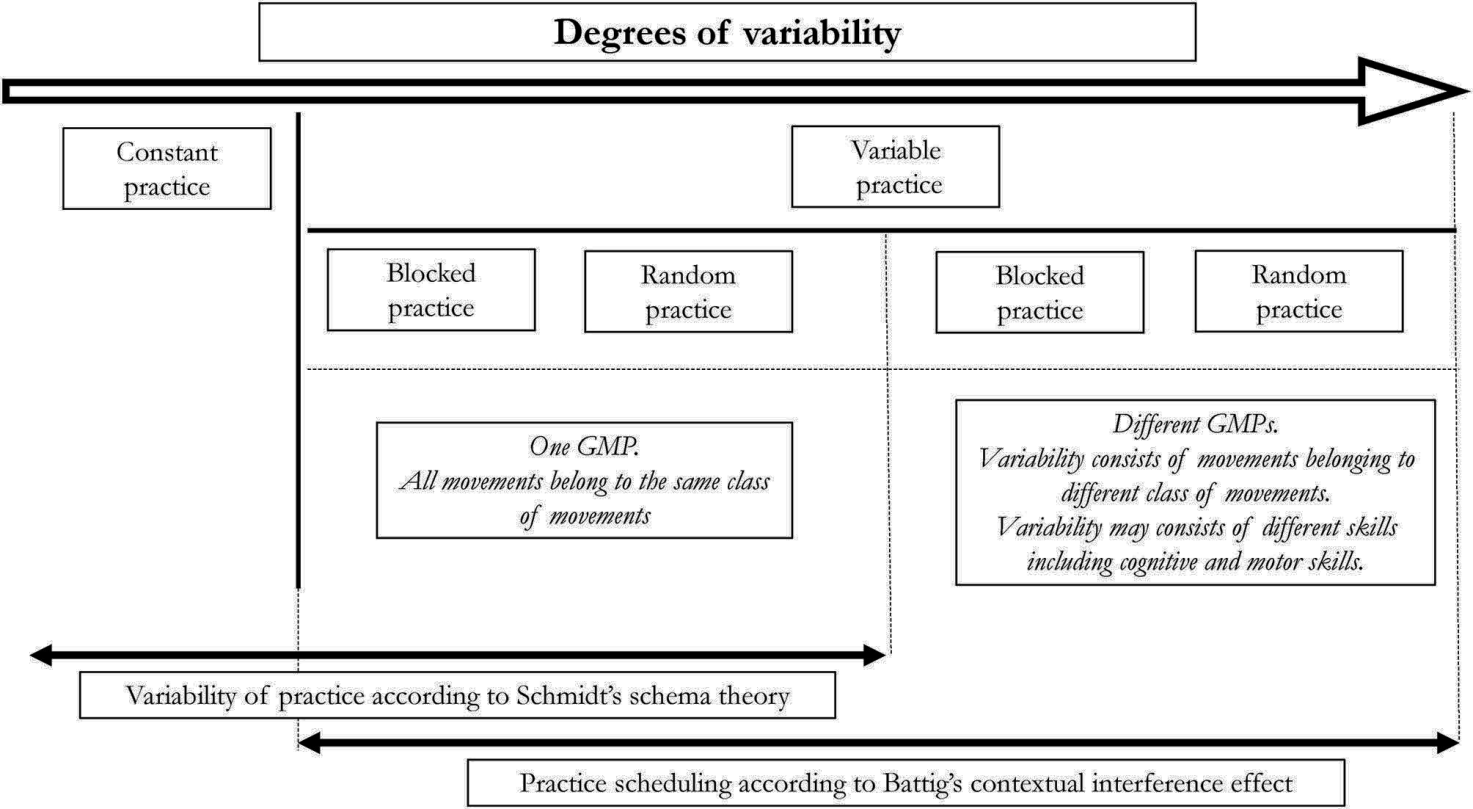
Degrees of variability from the least variable practice, i.e., constant practice, to the most variable, i.e., practice of different skills (e.g., cognitive and motor).

Both schedules, i.e., random and blocked, refer to the variable practice. However, they refer to different degrees of variability (Van Rossum, 1990) (see Figure 2). It is rational to assume that blocked practice is less variable than random. In both of them, although, individuals practice at least two tasks.

Furthermore, there is a substantial difference between variability of practice effects grounded in Schmidt's schema theory and variability of practice described by the contextual interference effect. According to Schmidt's schema theory, variability refers to a practice involving several variations of motor skill, governed, and executed by the same GMP, i.e., belonging to the same class of movements. On the other hand, the contextual interference effect can be found not only for motor skill variations controlled by the same GMP but also for variation of skills controlled by different GMPs (Magill and Hall, 1990). We could therefore argue that there are different levels of variability depending on whether the randomization consists of variations of the same motor skills (same GMP) or variations of different skills (different GMPs) (Figure 2).

Another term used in experimental psychology and cognitive neuroscience is task switching. These terms, contextual interference and task switching, are not used interchangeably. They also are attributed to different sources. Contextual interference comes from Battig (1966) and task switching approach from Jersild (1927). However, there are quite a lot of similarities between these two approaches. “*In task-switching experiments, participants perform a discrete task on each trial. On some trials the task changes (switch trials), and on others it does not (repeat trials)*” (Kiesel et al., 2010) (p.849). One can notice that repeat trials are analogous to blocked order whereas switch trials to random order.

### Dynamical Systems Theory

The problem of variability of practice is also addressed in dynamical systems theory. According to the dynamical systems theory, individuals adapt their movements to efficiently act in a complex environment (Davids et al., 2008). Variable practice helps to learn how to interact with the environment. The theory assumes that learning is a dynamical non-linear process when movement with unstable relationships between systems changes into a stable one. The systems stable state is invariant, i.e., it returns to a stable state after it is perturbed (Magill and Anderson, 2017). The states in which the systems are steady stable are called attractors (Davids et al., 2008). According to dynamic systems theory, movements can be characterized by order and control parameters. Order parameters enable to distinguish a movement with one set of coordination variables from another with a different set (Magill and Anderson, 2017). We could say that the order parameters are analogous to invariant features in Schmidt's schema theory. They define movement or a class of movement. We use them to distinguish and recognize movements from different classes of movements. On the other hand, control parameters can change enabling to modify/adapt movement to the environmental requirements. They could be compared to parameters in Schmidt's schema theory. However, unlike in schema theory, systematic changes in control parameters may lead to changes in stable states, i.e., one stable state may change into another stable state. According to the dynamical systems theory, the stability of the system emerges under environmental, organismic, and task constraints (Newell, 1986). These constraints act as limits or boundaries of the systems. Every movement is executed and produced within given limits. These limits may change depending on the systems interrelationships, however, the goal of the motor task still can be achieved.

Similarly, variability of practice as well as variability in performance (trial to trial) is at the core of the dynamical systems theory. The theory's proponents assume that individuals can achieve a goal using different coordination patterns. This concept is called degeneracy (Davids et al., 2007). The variability in performance is therefore, associated with different coordination patterns, yet allowing individuals to achieve a goal–the motor task outcome. Moreover, variability in performance is an immanent characteristic of movement in skilled individuals. It is because they are able to free the structure of movement, e.g., change their limbs' relationships although the limbs are still complementing each other, adjusting their particular movements to achieve a given task outcome.

The concept of degeneracy is also used when referring to the variability of practice since it allows an individual to adapt movement to different constraints. The dynamic systems theory assumes that each person is different and the environmental context is constantly changing, therefore, degeneracy permits a flexible adaptation to the new context. Consequently, it is essential to practice and learn while manipulating task constraints as it prepares an individual for new situations with different task and environmental constraints as well as it “trains” systems to adapt.

As one may notice, variability of practice is at the core of both approaches: motor program and dynamical systems theory. Both theories emphasize the role of variability of practice as a tool for either the development of schemata or increasing systems adaptability due to changing tasks and environmental constraints.

### Information Processing

In both the above described theories, information processing plays an important role. However, given they utilize different approaches to information processing, a short description should be provided.

In dynamic systems theory, information processing, specifically visual information processing, assumes that visual information is automatically processed in the way it constraints the motor system to produce an efficient movement (Gibson, 1979; Scully and Newell, 1985; Davids et al., 2008). This theory is called the dynamic theory of visual perception. It questions the need for coding and storing symbolic information in memory. This assumption is one of the fundaments of the dynamic systems theory. However, Gibson's theory of direct perception was doubted by Pizlo (2008). According to Pizlo, 3-dimensional shapes reconstructed in our brain from 2-dimensional images on our retina cannot be directly processed without additional information stored in our memory. The inverse problem and its elucidation by Pizlo (2008) eventually falsified Gibson's dynamic perspective on visual perception. Therefore, the classical theory of information processing much better explains the decision-making problems.

**Figure 3:**
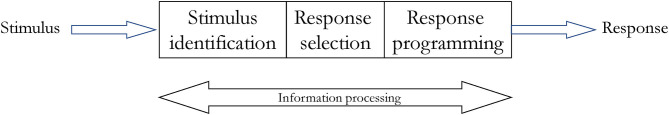
Three stages of the classical information processing model.

The so-called “classical” (Schmidt et al., 2018) information processing model assumes that the whole process can be divided into distinctive stages. The stages were recognized based on reaction time (RT) measures. The original idea of studying information processing using RT was introduced by Donders (Donders, 1868) and developed further as a chronometric approach (Sternberg, 1969; Posner, 1978). The classical information processing model consists of three stages: stimulus identification, response selection, and response programming (see Figure 3).

At the first stage, i.e., stimulus identification, a stimulus is detected and recognized. During the recognition process, an identifiable pattern of the stimulus is extracted and then processed as a meaningful one. At the second stage, i.e., response selection, a decision what to do in response to the recognized pattern of a stimulus is made. This is a decision-making process, absorbing cognitive resources, time-consuming, and energy-consuming. At the response programming stage (Henry and Rogers, 1960), the third and last stage of the classical information processing model, an individual has to translate the selected in the previous stage idea (an abstract idea of what to do) to specific and realistic commands for the motor system.

**Figure 4:**
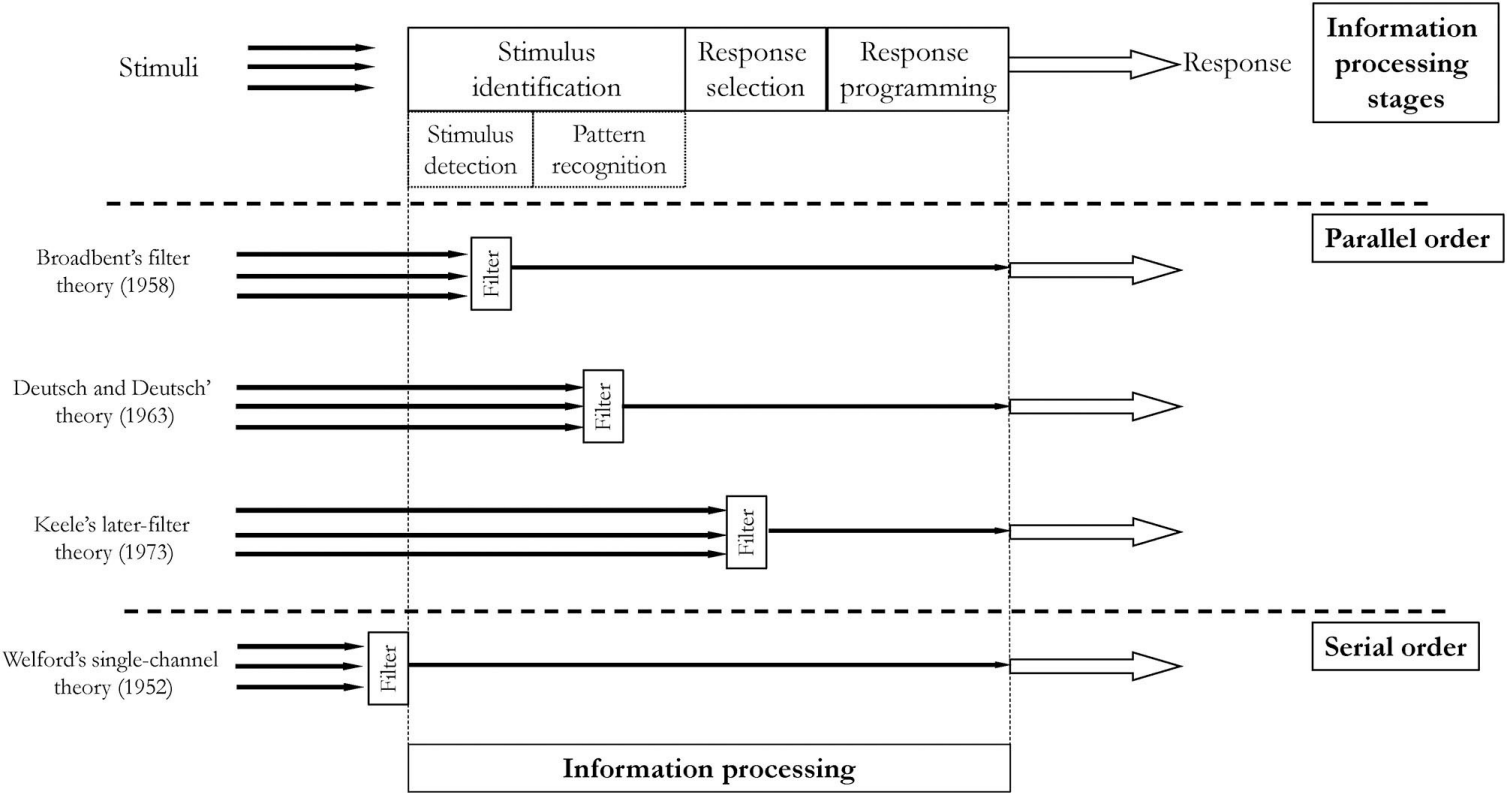
Serial and parallel order according to various theories of information processing.

There are a few variations of the classical model. They differ in the way multiple stimuli are processed. Many closely spaced stimuli may cause a delay in the information processing of the second stimulus–the total RT of the second stimulus is prolonged. This phenomenon is called the psychological refractory period (Telford, 1931). The variations of the classical information processing models address the delay issue, proposing places where “filters” holding on the latter information while processing the former are located. The differences between these models refer to whether the stages are organized in a parallel or serial order. Another difference between variations of the model refers to the place where so-called bottleneck occurs (Figure 4). Both of these aspects, i.e., order and limited capacity of information processing (bottleneck), are related to attention. i.e., the ability to consciously (awarely) perform perceptual, cognitive, and motor skills at the same time. The limitations in information processing are one of the characteristics of attention.

In a simple reaction task, the reaction time (RT) for one known stimulus, with one possible response, takes about 200 ms (Hick, 1952; Henry and Rogers, 1960). The first stage of information processing, i.e., stimulus identification in a simple reaction task, takes about 30 ms. Response selection, the second stage, takes about 55 ms (Schmidt et al., 2018). However, this stage has a different time duration depending on the number of choices an individual has while deciding what to do. Although the duration of this stage may slightly differ in individuals, according to Hick, every additional choice increases the reaction time by about 120–200 ms (Hick, 1952; Hyman, 1953; Proctor and Dutta, 1995). The linear relationship between the number of choices and the reaction time is called Hick's law, or sometimes Hick-Hyman law (Proctor and Dutta, 1995). Some authors claim that this relationship is not linear. Seibel (1962, 1963), for example, noted that when the number of stimuli to be screened increases from one to 32, the RT increases as well by ~20–30 ms. The number of 32 stimuli to be screened is equal to 4 bits of information–a measure of uncertainty (Attneave, 1959). Seibel (1963) also noticed that this increase does not follow a linear relationship, and increasing the number of stimuli to be screened from 31 to 1,023 does not increase the RT. We may assume, however, that increasing the number of possible choices increases RT by at least 20–30 ms up to 120–200 ms as originally noticed by Hick. The third stage of information processing is response programming. This stage takes at least 120 ms, although its duration is affected by the complexity of a response (movement). The relationship between movement complexity and RT was firstly presented by Henry and Rogers (1960) and was corroborated in many following studies (Klapp, 1995, 2003). The more complex is the response, the longer it takes to prepare it, i.e., RT is longer.

### Attention

Limited capacity of attention is unquestioned these days (Neumann, 1996). Some of the first theories of attention emphasized its limitation as one of the most representative features. According to one of the theories, individuals struggle with performing several things at one time, because the information processing stages are organized in a serial order, i.e., only one piece of information can be processed at one moment. This theory is called single-channel theory (Welford, 1952). On the other hand, parallel order theories assume that at a specific information processing stage, there is a bottleneck, where the information is filtered for further processing. This theory was called filter theory (Broadbent, 1958) or bottleneck theory. Variations of the bottleneck theories differ in terms of placing the bottleneck at different information processing stages. Some theories claim that the bottleneck (filter) occurs at the stimulus identification stage, either at detection (Broadbent, 1958) or recognition phase (Deutsch and Deutsch, 1963) (see Figure 4). Other theories, e.g., Keele's late-filter theory (Keele, 1973), placed the bottleneck at the response selection stage.

## Information Processing and Variability of Practice

The keywords “reaction time” or “response time” AND (Boolean operator) “variability of practice” searched in databases such as EBSCO Academic Search Complete, APA PsychInfo, Medline, SPORTDiscus yielded only nine relevant publications (excluding two duplicates). Within these nine, there was one dissertation abstract and one conference proceeding. Only five of them were related to motor learning. Although searched terms appeared in the abstracts, the papers did not specifically focus on information processing and decision making and variability of practice but rather used reaction or response time as a measure of performance or learning improvement. Search was performed on 13 November 2020.

Another search with keywords “decision making” AND “variability of practice” did not generate any related publications. Two relevant publications were found after searching the terms “information processing” AND “variability of practice” (search on 15 January 2021), i.e., by Chua et al. (2019) and by Deutsch and Newell (2004). Both of them are cited in this paper.

### Information Processing in Constant Practice Conditions

To specify how different practice conditions, affect decision making, we need to consider how information can be processed in a single reaction and discrimination task under constant practice conditions. Very often, when referring to constant practice conditions, the term “specificity of practice” is used, as opposed to “variability of practice” when talking about variable practice conditions.

In constant practice conditions, only one set of parameters is assigned and one GMP is used. Therefore, no choice reaction task can be introduced in practice as it would require changing the parameters and perhaps GMPs. As a result, there are only two possible ways of organizing constant practice. We can either:

1. utilize a simple reaction task; or
2. utilize a discrimination task.

**Figure 5:**
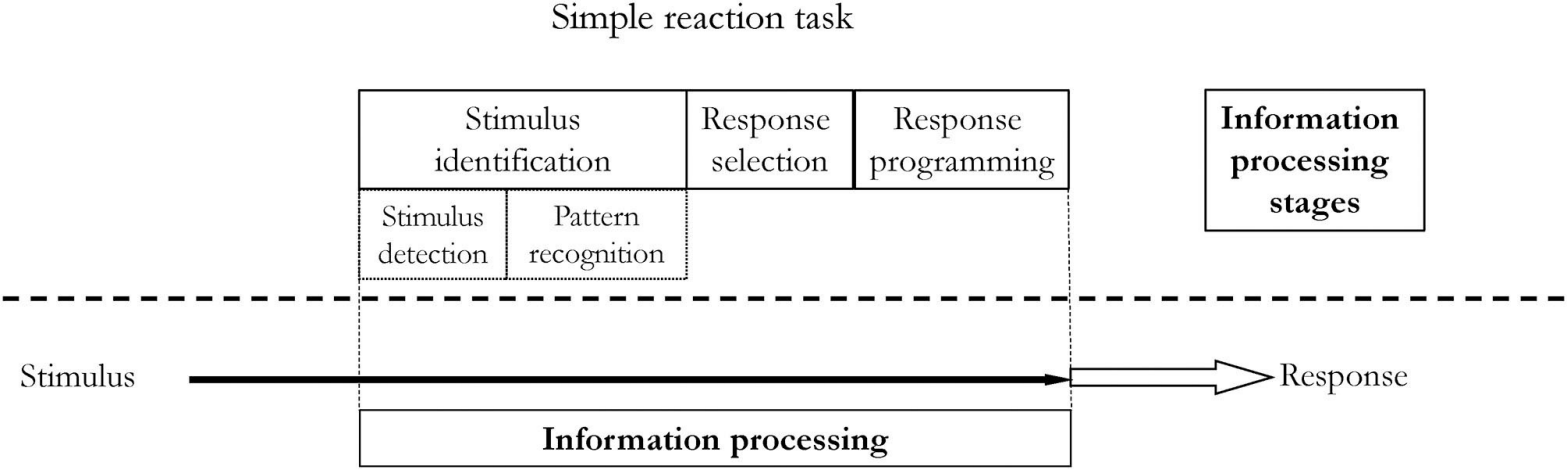
Information processing in a simple reaction task under constant practice conditions.

**Figure 6:**
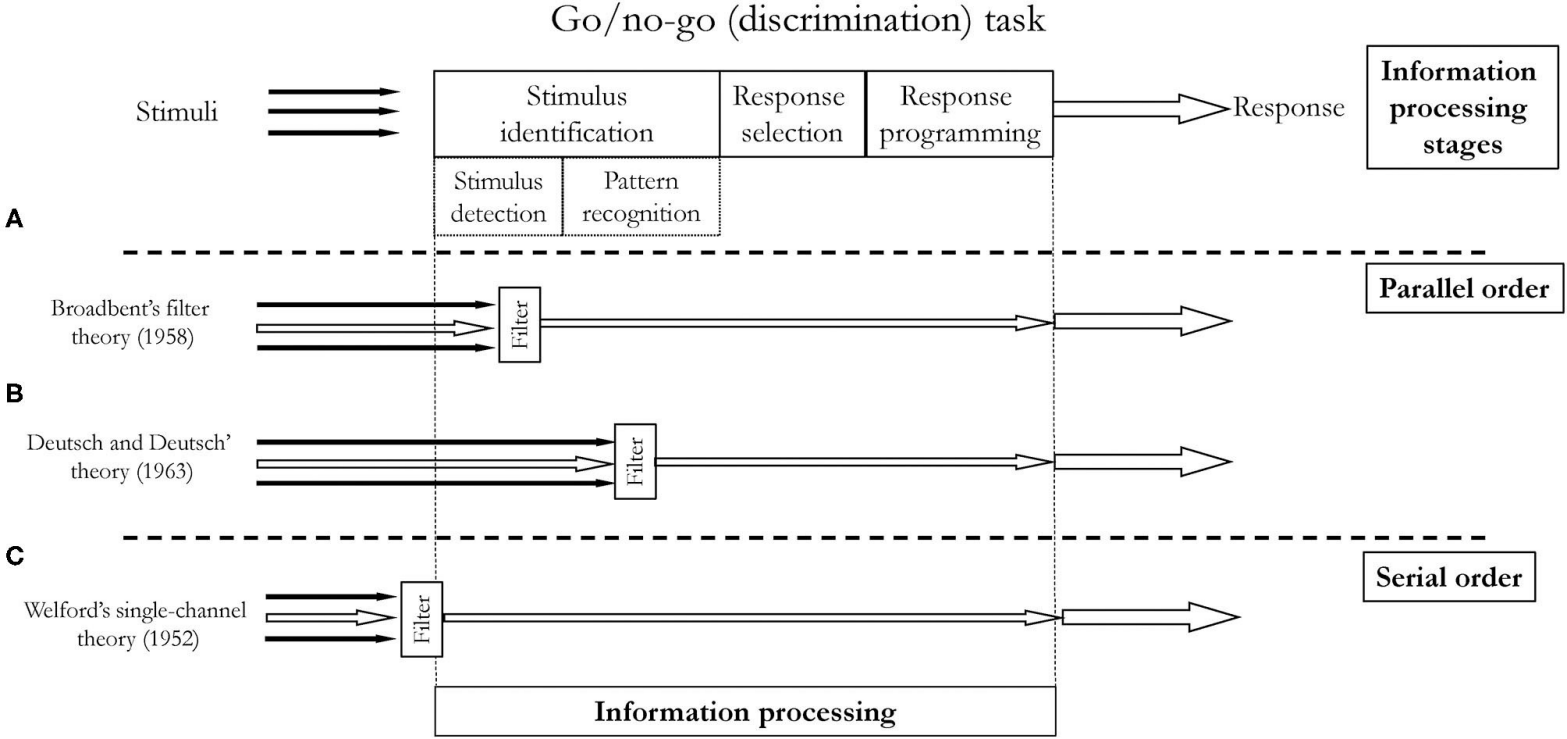
Information processing in go/no-go reaction task under constant practice conditions. Unfilled arrows indicate the stimuli that have to be recognized as “go” stimuli. They are processed until the ultimate response production. There is only one possible response since, in constant condition, only one variation of skill is practiced. Three hypothetical processing are presented: **(A,B)** in a parallel order where the filter is located at the stimulus identification stage (detection or pattern recognition phase); **(C)** in a serial order.

The most obvious information processing model in constant practice utilizing a simple reaction task is presented in Figure 5. It consists of one stimulus and one corresponding response. In the presented situation, no matter whether the information processing is parallelly or serially ordered. It will not affect the reaction time and decision making. However, constant practice may also refer to situations when there is only one possible reaction which has to be produced in response to only one of the many stimuli present in our environment, i.e., in discrimination tasks (or go/no-go). These situations are presented in Figure 6. It is quite rational to assume that in real life, we are exposed to many stimuli, hence this situation is more accurate, e.g., there are many stimuli when we hunt, but only a specific one, a sight of a game, makes us to release a rifle trigger. Therefore, the RT will depend on how many stimuli we have to screen to recognize the appropriate one. While detecting and recognizing stimuli patterns during the first stage, we eliminate (drop off) redundant stimuli and process only one. An individual has to screen all stimuli and see if there is one triggering the planned response (movement). The process of discriminating one stimulus from the others may increase RT. Another possible situation is when there are more than one stimulus triggering the response (movement) and only one response. Perhaps the number of to-be-screened stimuli and the number of triggering stimuli may affect RT. However, it has not been confirmed yet. On the other hand, the more often an individual is exposed to a stimulus, the shorter is the stimulus identification stage, specifically the pattern recognition phase. Individuals become better, i.e., faster, at picking up the patterns of a stimulus. The influence of learning on pattern detection was firstly demonstrated by Chase and Simon in chess players (Chase and Simon, 1973), however, this learning effect is observed across different tasks and skills (Causer et al., 2012; North and Williams, 2019; Williams, 2020).

We may also assume that in constant practice conditions, the non-triggering stimuli would never achieve a later stage, e.g., the response selection stage, as proposed in Keele's later stage theory (Keele, 1973). Therefore, any increase in RT could be associated with the longer stimulus identification stage rather than with response selection or response programming. Yet, there is only one possible response with one set of parameters ready to be used.

Another problem relates to the similarity between stimuli to be recognized. We can only speculate how similarity could affect information processing and RT and decision making. If an individual is exposed to many stimuli of which only one triggers the response, and the stimuli are not very similar one to another, then it should be much easier to “screen” them and recognize stimuli patterns. Recognition could allow to either eliminate the no-triggering stimuli and accept for further processing only the appropriate one. On the other hand, if the stimuli are very similar one to another, then pattern recognition should be more meticulous and would take more time. Would such in-depth screening increase the RT? Again, there is no study simply answering this question. We could draw an analogy from the CI effect in which one of the sources of interference is the nature of the practiced tasks. The interference is higher when the performed tasks are more similar one to another (Shea and Morgan, 1979; Battig and Shea, 1980). The more similar tasks which are randomized, the better learning and transfer. However, the high CI caused by the increased similarity of performed tasks hinders performance. The most substantial difference, however, between constant practice with many similar stimuli and only one response and contextual interference with similar tasks randomized is that in randomized practice the possible responses have to be screened at the response selection stage. There is no such need in constant practice conditions. Moreover, the programming of the movements is different for specific tasks used in random order (Shea and Zimny, 1983; Lee et al., 1985). It is much more demanding to assign parameters in random order than in blocked order. We may assume that the easiest programming occurs in constant practice. There is only one possible response and one set of parameters to be assigned, hence not additional time is needed to reprogram a response. At this point, we can only speculate whether the similarity between the stimuli to be screened to find the triggering one increases RT and whether this increase deteriorates performance but enhances learning and transfer analogically to the high CI effect.

The question about the relationship between similarity of the stimuli to be screened and RT has a practical implication as well. E.g., one could think about training procedures or a competition design in which the environment would involve several similar stimuli and as a result RT would be longer. Let's imagine a training drill in tennis, in which a tennis ball machine throws balls at the baseline or a sideline. A player is asked to hit the ball which would land inside the court but do not hit a ball which would land outside of the court. Hence, the response would be (almost) the same: e.g., forehand. It would be much easier to recognize which ball is which if they are quite distinct in their flight, i.e., if they would land quite far from the court lines or one from another, either in or out of the court. However, the more similar they would be, i.e., they would land closer to the lines or just outside the lines and closer one to another, we could assume that perceiving the important pattern of the flying ball could take more time and as a result would increase RT. The practice itself, with the balls landing very close one to another could be more demanding and difficult compared to the practice with balls landing quite far. A trainer would therefore consider that such practice may increase RT and as a result decrease and reduce the available time for movement performance. Would a player have enough time to perform an efficient and technically correct movement? As van Rossum noted (Van Rossum, 1990) when “*a movement of longer duration is executed, the possibility exists to adapt the movement in the course of its execution*” (p. 391). Limiting time for movement execution may affect its outcome as there is no time for parameters adjustment in response to environmental requirements. Although we assume that in constant conditions there is no need for parameters adjustment, we have to also consider that there is always such a need due to the trial-to-trial variability.

Related finding was reported by Czyż et al. (2019) in a paper on specificity of practice. Movement, free throw in basketball, practiced in constant conditions had an overall shorter duration at the end of the practice sessions than the same movement but practiced in variable practice. Participants in their study practice free shots in variable (5 or 7 different distances) or constant (one distance) conditions. Variability concept in their study was drawn from the schema theory, i.e., movement variations belonged to the same class of movement (the same skill) and were thought to be operated and executed by the same GMP. Unfortunately, the authors did not differentiate the reaction and movement time, so we cannot not say whether the longer response time was due to the longer information processing (RT) and what stage was accountable for it. It is also probable that the longer response time was due to the closed-loop feedback responsible for movement adjustment in a different parameter setting required at different distances. As Khan and colleagues stated “*Movement time does not vary with response complexity in a straightforward manner, and understanding the factors that influence this relationship has remained a challenge for researchers interested in the processes associated with movement preparation and execution*” (Khan et al., 2010) (p. 97).

**Figure 7:**
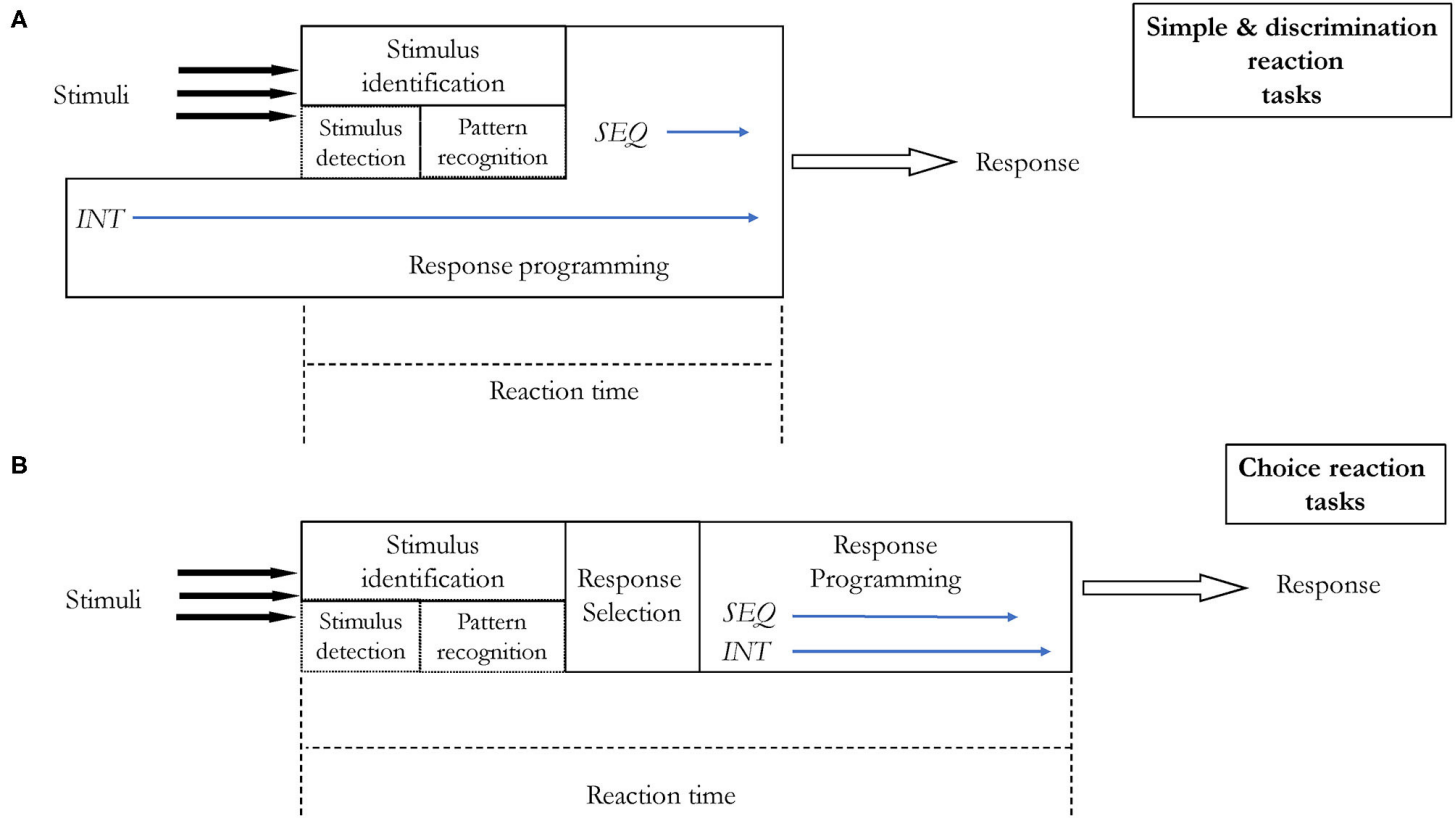
Information processing with two distinct processes within the response programming stage proposed by Klapp (1995, 2003): *INT* and *SEQ*. *INT* is responsible for programming internal features of individual elements while *SEQ* for scanning of an abstract initiation time of a chunk without reference to the content. *INT* in simple reaction time tasks **(A)** can start before a stimulus is present—response can be preprogrammed. In choice reaction time tasks **(B)**, preprogramming is not possible—therefore *INT* increases the overall RT. In simple reaction time tasks no response selection stage occurs.

Complexity of a response affects RT as originally observed by Henry and Rogers (1960). Another important aspect of this finding was discovered by Klapp (1995, 2003) in experiments utilizing Morse code responses, i.e., in cognitive-motor task. Klapp noted that the RT increased in a simple reaction task when the number of elements to be coded increased. On the other hand, in the choice reaction time task, the RT increased when the duration of sequences was longer. Klapp proposed that within the response programming stage there are two distinct processes. The first, called *INT*, is responsible for programming internal features of individual elements (e.g., its duration). An analogy to parameter assignment in Schmidt's schema theory is here justified. The second process, called *SEQ*, is responsible for scanning the time frames of sequences, i.e., for localizing the initial starting points of each sequence. Klapp concluded that in simple reaction tasks, *INT* occurs prior to the programming stage. On the other hand, preprogramming is not possible in the choice reaction time tasks as an individual has to “decide” which response to choose and eventually program it. Hence, both processes, *INT* and *SEQ* befall during RT (see Figure 7). However, *INT* takes much longer than *SEQ*, therefore, RT in choice reaction task is longer than in simple reaction task. This effect was confirmed in subsequent studies (for references see: Khan et al., 2010). The findings by Klapp have an important practical implication too. Given that in simple reaction tasks the programming stage, at least partially, can be initiated prior to the stimulus presence, the RT will be shorter. In tasks like, e.g., ball hitting in baseball, where there is limited time for information processing and movement, one could manipulate learning conditions to facilitate either movement performance or information processing. If an individual has a problem with movement, then the learning could be organized in a constant manner. The response programming stage in such a case, could start before the ball is thrown, decreasing RT and as a result increasing the available time for movement performance. And vice versa, if we would like to improve information processing, we could organize the learning in variable conditions, preferably utilizing choice reaction tasks.

### Information Processing in Variable Practice Conditions

**Figure 8:**
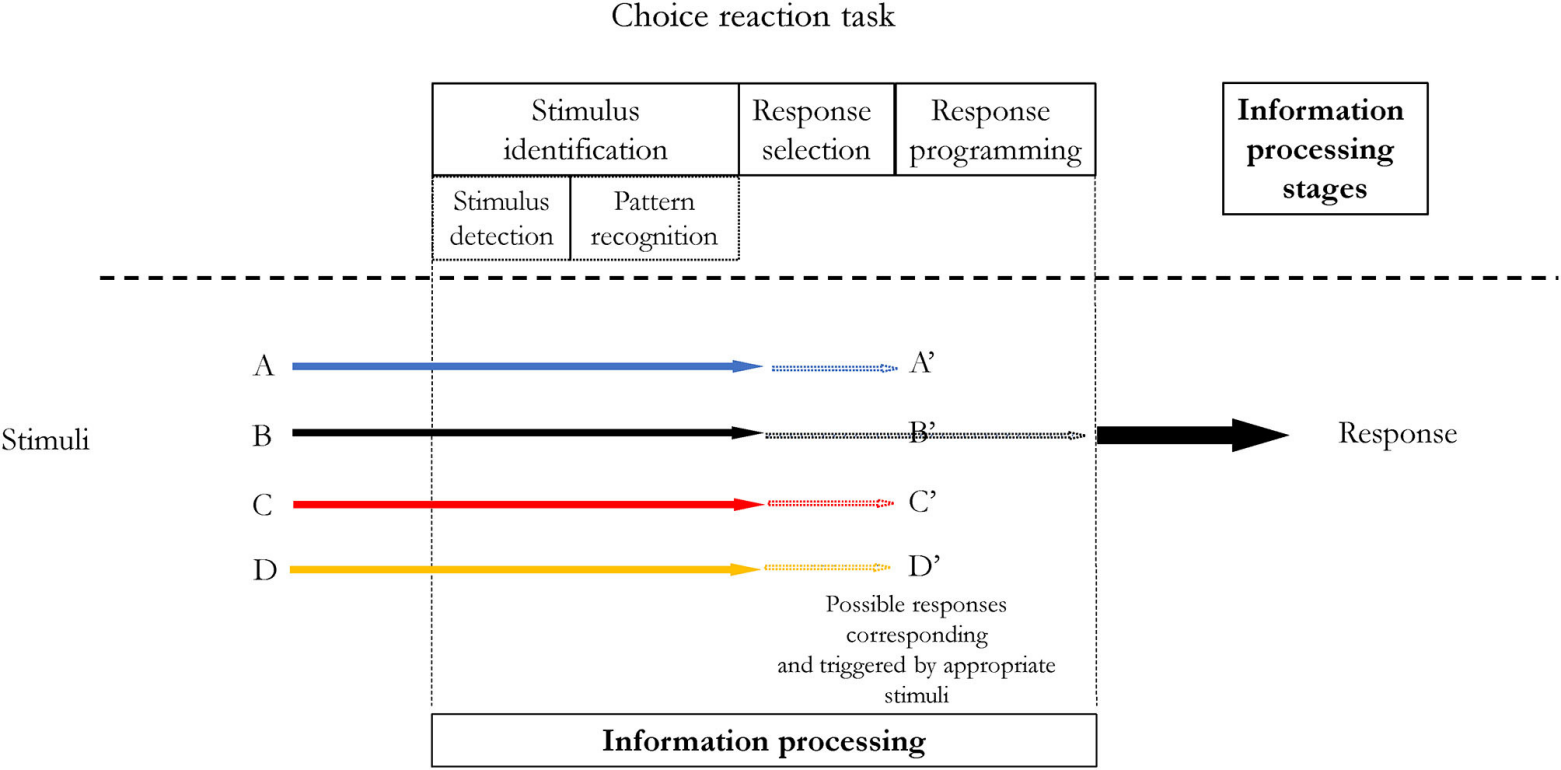
Information processing in choice reaction task under variable practice conditions in a parallel order. An individual exposed to four stimuli (A, B, C, and D–solid lines) can trigger the corresponding responses (A', B', C', and D'–dashed arrows). The B' response is chosen and processed further through the programming stage.

Even more complicated situation is in variable conditions practice. Generally, there are more “stimulus-response” pairs. i.e., an individual has to recognize an appropriate stimulus and pair it with a corresponding response (see Figure 8). However, this is not specifically the only situation a variable practice can be organized in.

Variable practice can also consist of simple reaction tasks, like the ones presented in Figures 5, 6, 8, however, these tasks can change from trial to trial producing different movements in response to different stimuli. This problem is directly related to practice scheduling. It seems that variability of practice has to be inherently considered in practice scheduling. The variable practice may be organized in the following ways:

1. it can consist of simple reaction tasks or discrimination tasks in a blocked order;
2. it can consist of simple reaction tasks or discrimination tasks in a random order.
3. it can consist of choice reaction tasks organized in a blocked order;
4. it can consist of choice reaction tasks organized in random order;
5. It can be a mixture of any of the above.

In the first two schedules, information processing could be similar to the information processing in constant conditions. In this case, as long as the same task is performed and practiced, similar to constant conditions, mechanisms apply. The only difference and perturbation could be observed while switching from one task to the other. There is no reason to assume that the stimulus identification stage in variable conditions takes longer than in the constant practice if the number of stimuli to be recognized is the same. Although, this statement needs empirical support. Additionally, in random order, a bigger load on attention could be observed as an individual would have to switch from one program to the other (interference effect). Increased attention demand in a random schedule may be associated with planning an upcoming response–movement (Wright et al., 2016). The random practice is also more effortful information processing (Chua et al., 2019). It may be attributed to the changes of external focus (changes in the task goal). i.e., the external focus frequently changes in random practice (Chua et al., 2019).

Information processing could be more complicated when practice would involve a choice reaction task (situations 3–5). The RT will be increased due to the increased response selection duration (Hick, 1952; Hyman, 1953; Seibel, 1962, 1963; Schmidt et al., 2018). Additionally, unlike in constant practice conditions utilizing simple or discrimination RT tasks, *INT* process within the response programming stage (Klapp, 1995, 2003) cannot start before a stimulus is presented. The *INT* process will occur within the RT, increasing its duration.

Apparently, the best documented relationship between RT and variable practice conditions is the one based on the contextual interference effect. Unfortunately, we lack research on RT and variability of practice from the schema theory perspective in general. As Broadbent et al. (2019) stated “*Those examining the training of anticipation and decision making have generally not addressed the structure of practice*” (p. 290). They emphasized that the majority of studies focused on the randomization effect, i.e., contextual interference effect, rather than on variability of practice from the Schmidt's schema theory perspective. And yet, the number of these studies is quite limited. For example, in two studies, Broadbent et al. (2015, 2017) focused on CI effect and decision making process. In both studies, participants decreased decision time in transfer tasks following random practice. These studies generally support previous findings on information processing and confirm CI effect as described by Battig (1966, 1972). However, these do not say much about the mechanisms of improvement.

On the other hand, there is “a switch cost” in reaction time (RT) and error rates while task switching (Kiesel et al., 2010). Delayed responses noticed in task switching procedures are probably caused by higher working memory load or executive control processes. However, there is no conclusive evidence on the latter one (Kiesel et al., 2010) and there is no direct linkage between these two approaches (contextual interference and task switching).

### Information Processing in Variable and Constant Practice–Practical Implications

Although there is still a gap in research on variability and specificity of practice and information processing, we can advance some theoretical and practical hypotheses. Most of the motor learning books for either practitioners or academics suggest that implementing a higher degree of the variability, i.e., high contextual interference, in practice may enhance learning and transfer (e.g., Boyce et al., 2006; Vickers, 2007; Coker, 2017; Magill and Anderson, 2017) also in terms of faster and more appropriate reaction, i.e., in decision making. Not many though, mention that there are studies questioning the positive effect of high contextual interference on retention and transfer (for review see: Brady, 2004; Lee, 2012). Moreover, the meta-analysis of contextual interference by Brady (2004) shows that the effect size for contextual interference in applied studies was small (Cohen's *d* = 0.19). On the other hand, there are no studies explaining how practicing in constant conditions would affect both RT and decision-making process. This problem was broached by Broadbent et al. (2019), however it has not been broadly addressed yet.

Another problem related to practice variability and information processing is that there is inconclusive support for enhanced learning and transfer in timing tasks following variable practice as predicted by Schmidt's schema theory (Van Rossum, 1987, 1990). Perhaps it is associated with the motor tasks used in studies on variability of practice. Kerr noted (Kerr, 1982) that in the research on variability of practice hypothesis, the most used tasks were closed skills, with fixed environments with some intertrial variability. As a result, Kerr argued that individuals were focusing more on movement performance than on decision making, i.e., participants focused more on the type of movement one has to make to achieve a goal. As it was already discussed, in simple reactions tasks, i.e., based on closed skills, the response programming stage can start well ahead of stimulus presentation. Considering Klapp's findings (Klapp, 1995, 2003), one could hypothesize that the most conspicuous differences between information processing in constant practice conditions and variable practice conditions will be when comparing simple or discrimination reaction tasks in constant practice and choice reaction time tasks in variable practice conditions. The difference between the RT in these tasks would be due to the present response selection and increased response programming stages.

From a pragmatic point of view, practitioners could think about the consequences of practice conditions manipulation on information processing. Firstly, they can use different reaction time tasks. Simple and discrimination reaction tasks would decrease RT, since there will be no response selection stage and the response programming stage can start before a stimulus is presented. It can be assumed that this type of practice would facilitate response programming and movement performance as attention capacity could be used mostly to execute and govern movement. Secondly, when the bigger emphasis has to be put on attention load and bigger cognitive effort, then training should consist of choice reaction tasks. Given there is a widespread belief that the more cognitively demanding practice is better for learning (Vickers et al., 2004; Vickers, 2007), some trainers, coaches would like to increase cognitive effort to enhance retention and transfer. In their practice, they could include choice reaction tasks in random order since the bigger attention load is noticed in practice scheduled this way. Hence, coaches could progress the practice difficulty through including choice reaction time tasks in blocked (lower cognitive effort) or in random order (bigger cognitive effort).

### Information Processing and Variability and Specificity of Practice–Possible Future Directions

Apparently, the relationship between information processing and practice variability and specificity is not well-recognized yet. The already conducted studies focused more on the higher degree of variability, i.e., on contextual interference, randomized either with variations of the same skills or different skills. Little is known about how practice in constant conditions affects information processing and whether it enhances learning and transfer. This problem should be addressed in future studies.

There is a lack of studies comparing information processing in constant practice conditions including simple or discrimination reaction tasks and variable practice conditions consisting of choice reaction time. This comparison may have two-fold benefits. Firstly, it would extend our knowledge about the mechanisms underlying variability and specificity of practice. On the other hand, it could help to support the original variability of practice hypothesis.

**Figure 9:**
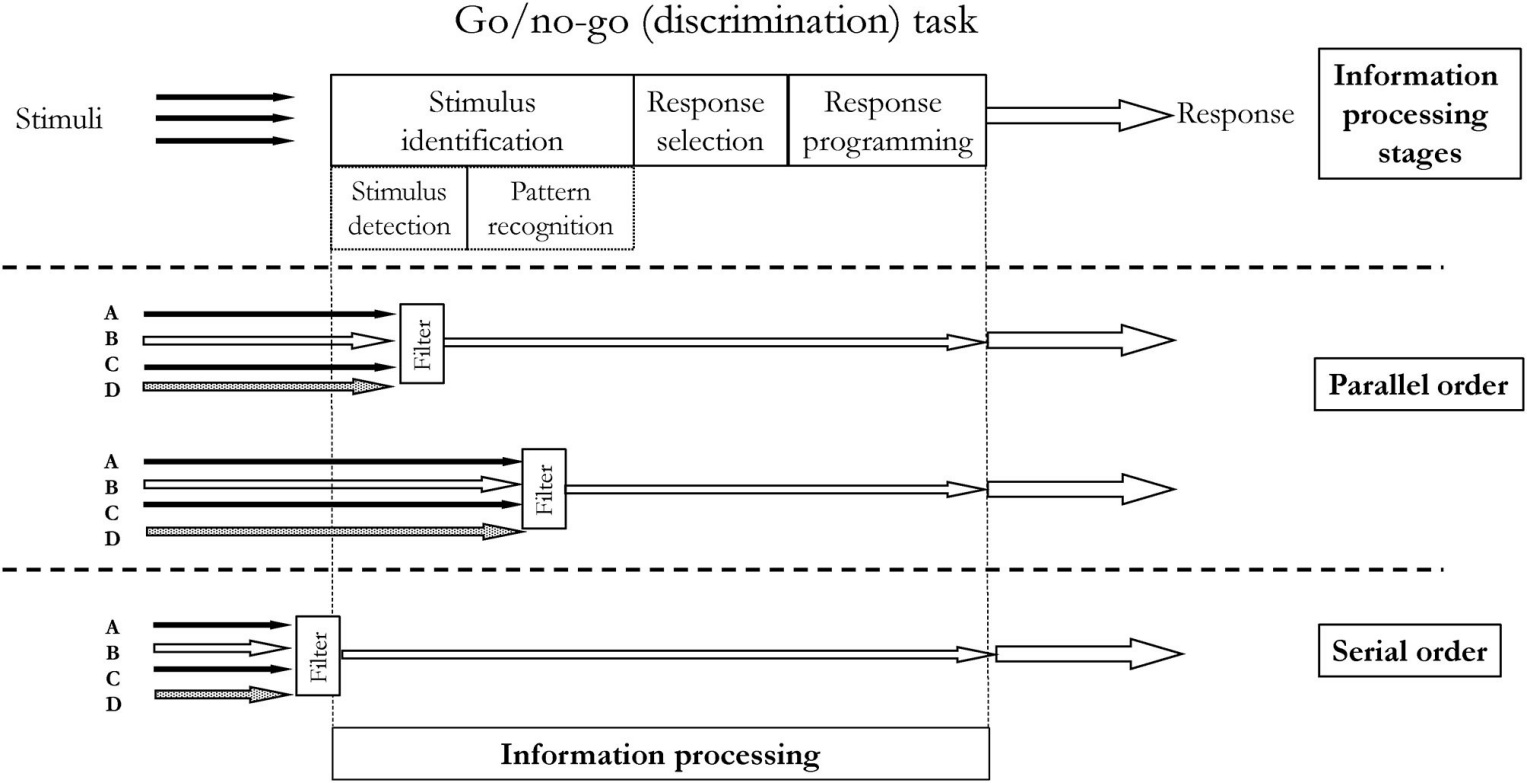
Information processing in discrimination reaction task with one possible response and more than one stimulus triggering the response (movement). Response can be initiated by either stimulus B or D.

Another problem not well-addressed in the research, yet relates to the number of stimuli triggering one possible response as presented in Figure 9. If there is only one possible response and a few stimuli which could trigger it, how “screening” of the stimuli would affect information processing and RT? Moreover, there are no studies investigating how the similarity of stimuli an individual is exposed to affects information processing and decision making. Although some analogies from studies on classical and instrumental conditioning (Anderson, 2000) could be drawn, there is a need to determine what is the influence of stimuli similarity on information processing. In conditioning, the more similar stimuli are, the more difficult is to learn how to differentiate (discriminate them). Future studies could therefore determine whether similarity of the triggering stimuli affect RT and decision making.

Lastly, some of the terms, such as closed and open skill (Poulton, 1957) may be directly associated with simple and discrimination reaction tasks or choice reaction tasks accordingly. Although these tasks do not run out all possible tasks within these skills, information processing in closed skills can be simple reaction tasks or discrimination tasks only. Choice reaction tasks cannot be closed skills, since the skill is performed in an unpredictable environment. As Poulton stated, closed skills are named so “*because the performance can be carried out successfully without reference to the environment*” (Poulton, 1957)(p.472). Moreover, in closed skill, there are no external requirements or an individual can make predictions about the possible environmental requirements.

### Limitations of the Review

Given there are quite a few different terms used when referring to variability of practice, perhaps a review using other key words would bring more results. There are already thousands of publications on information processing utilizing motor tasks. Perhaps some of them deal with the variability of practice problems, although not explicitly naming it so.

Another problem relates to the approaches and paradigms used when interpreting and studying information processing. For example, another theoretical approach is used to interpret the variability of practice benefits and disadvantages. The task switching procedure, widely described in experimental and cognitive psychology, serves as a base for theorizing about executive control mechanisms. Although quite conspicuous analogy to the random and blocked conditions in variable practice, studies on task switching and repeated practice are not widely cited in the motor learning field and *vice versa*. It is surprising since in many task switching experiments motor tasks are used and the paradigm used to define the characteristics of body movements prior to the time of movement completion was originally proposed for motor programming (Rosenbaum, 1980). Therefore, a review with a deeper and more thorough emphasis on task switching studies should be considered while discussing variable practice conditions. They can extent already existing knowledge in motor learning field.

## Author Contributions

The author confirms being the sole contributor of this work and has approved it for publication.

## Conflict of Interest

The author declares that the research was conducted in the absence of any commercial or financial relationships that could be constructed as a potential conflict of interest.
